# Ultrasonographic evaluation of deep vein thrombosis related to the central catheter in hemodialytic patients

**DOI:** 10.1186/s13089-021-00252-4

**Published:** 2022-01-10

**Authors:** Ana Luisa Silveira Vieira, José Muniz Pazeli Júnior, Andrea Silva Matos, Andreza Marques Pereira, Izadora Rezende Pinto, Letícia Esteves de Oliveira Silva, Letícia Siqueira Guilherme, Sofia Laura Archângelo e Silva

**Affiliations:** 1 Department of Point of Care Ultrasound, Barbacena Medical School, Minas Gerais, Brazil; 2 Department of Nephrology, Santa Casa de Misericórdia de Barbacena, Minas Gerais, Brazil; 3 Barbacena Medical School, Minas Gerais, Brazil

**Keywords:** Venous thrombosis, Hemodialysis, Ultrasonography, PoCUS, Medical students, Training, Dialysis, Ultrasound

## Abstract

**Background:**

Point of care ultrasound (PoCUS) is a useful tool for the early diagnosis of thrombosis related to the central venous catheter for dialysis (TR-CVCd). However, the application of PoCUS is still not common as a bedside imaging examination and TR-CVCd remains often underdiagnosed in the routine practice. The aim of this study was to investigate if a compression technique for the diagnosis of TR-CVCd blindly performed by PoCUS experts and medical students is accurate when compared to a Doppler study.

**Methods:**

Two medical students without prior knowledge in PoCUS received a short theoretical–practical training to evaluate TR-CVCd of the internal jugular vein by means of the ultrasound compression technique. After the training phase, patients with central venous catheter for dialysis (CVCd) were evaluated by the students in a private hemodialysis clinic. The results were compared to those obtained on the same population by doctors with solid experience in PoCUS, using both the compression technique and the color Doppler.

**Results:**

Eighty-one patients were eligible for the study and the prevalence of TR-CVCd diagnosed by Doppler was 28.4%. The compression technique performed by the students and by experts presented, respectively, a sensitivity of 59.2% (CI 51.6–66.8) vs 100% and a specificity of 89.6% (CI 84.9–94.3) vs 94.8% (CI 91.4–98.2).

**Conclusion:**

The compression technique in the hands of PoCUS experts demonstrated high accuracy in the diagnosis of TR-CVCd and should represent a standard in the routine examination of dialytic patients. The training of PoCUS inexperienced students for the diagnosis of TR-CVCd is feasible but did not lead to a sufficient level of sensitivity.

**Supplementary Information:**

The online version contains supplementary material available at 10.1186/s13089-021-00252-4.

## Introduction

Chronic kidney disease is considered a great challenge for Brazilian public health due to the continuous increase in the number of patients with the need of renal replacement therapy [1].

According to recent Brazilian data, about one hundred thousand patients are in dialysis treatment, 90% of them in hemodialysis therapy [2].

The patients with renal failure that evolves to the indication of hemodialysis need an appropriate venous access, which may be either a central venous catheter for dialysis (CVCd) or an arteriovenous fistula (AVF) [3].

Despite being safer for the patient, the creation of an AVF requires a costly and complex surgical procedure [2, 4]. Therefore, the use of CVCd is more frequent among those patients in hemodialysis, although it is burdened with complications that increase the morbimortality rates in this population, such as bacteremia, septicemia and thrombosis [4, 5].

The prevalence of thrombosis related to CVCd (TR-CVCd) is highly variable in the literature. Agraharkar and collaborators reported a prevalence of 2% [6], while Karnik and collaborators observed 63% [7]. The formation of a thrombus can generate severe systemic complications, such as pulmonary embolism [8], that is why early diagnosis and treatment are fundamental.

With regard to risk factors, the occurrence of TR-CVCd is related to conditions intrinsic to the patient (neoplasms, acquired or hereditary thrombophilia) or directly related to the catheter (insertion site, material, period of permanence and number of catheters inserted) [9].

The diagnosis of TR-CVCd in dialysis centers is hindered by the absence of symptoms in most cases [9]. In this scenario, the point-of-care ultrasonography (PoCUS) in the hands of the same clinician who assists the patient and performed as an extension of the traditional physical exam, has emerged as a promising diagnostic tool.

PoCUS is a relatively easy technique to learn, non-invasive, free of ionizing radiation risks, efficient and can be performed at the patient bedside, without significant additional cost to the treatment [10, 11].

Despite the benefits of PoCUS, only recently its use has spread in nephrology, most of the time focused to guide percutaneous procedures (dialysis catheter implants and renal biopsies) [11, 12] and to evaluate urinary tract diseases [10, 12].

To date, there are no studies published about the evaluation of TR-CVCd by nephrologists, using the PoCUS compression technique to diagnose vessel thrombosis in the internal jugular vein (IJV). In addition, the performance of the PoCUS examination by medical students trained for a short period of time to the diagnosis of TR-CVCd at bedside is not sufficiently explored.

The main aim of our study is to compare the accuracy of the PoCUS compression technique in the diagnosis of TR-CVCd of the IJV, with the well-established Doppler method. A second aim is to evaluate if a short and basic training on the compression technique can lead medical students without prior knowledge in PoCUS to diagnose TR-CVCd with good efficiency. The study also seeks to determine the prevalence of TR-CVCd in a standard Brazilian hemodialysis center.

## Methods

### Design of the study

An analytical observational cross-sectional study was performed for the ultrasound diagnosis of deep vein thrombosis in the IJV, in dialysis patients with central venous catheter implant for hemodialysis. The evaluation was performed independently by medical students, after a short training period, and by doctors experienced in PoCUS using the ultrasonographic technique of vein compression. The results were compared to those obtained blindly by other experienced doctors performing the color Doppler technique to confirm the diagnosis.

### Training of the students

The study team was composed of two students from FAME Medical School without prior knowledge in PoCUS and by three doctors with vast experience in the technique. The training of the students included: (a) first stage: basic explanation on fundamental physics principles of ultrasound, focused on the ultrasonographic evaluation of the IJV and the adjacent structures, totaling 90 min of training; (b) second stage: practical training to learn the vessel recognition technique and to acquire the compression technique on eight patients with and without deep vein thrombosis, totaling 120 min.

### Population

The study was conducted on patients on chronic hemodialysis followed in a dialysis clinic of the city of Barbacena, Brazil, that attends 172 dialysis patients. This study included all hemodialytic patients of both sexes, regardless of age group and comorbidities, with a history of CVCd implant in the IJV.

We excluded patients with a clinical history of deep vein thrombosis not related to a catheter implant, those with physical impossibility to examine the region of the IJV, or those who were making use of the central venous catheter at the time of the examination. We also excluded patients who did not sign the Informed Consent Form.

### Study protocol

Consulting the electronic medical records of the clinic, the medical students initially selected the patients with a non-tunneled catheter implant in the IJV. Information about demographic data, family history and comorbidities were collected by interviews, to characterize the sample profile.

The ultrasonographic examinations were performed using a portable ultrasound device (Terason t3000CV; Terason, Burlington, MA, USA) with a high frequency linear transducer (6–12 MHz).

The patients were examined during their hemodialysis session, in a semi-recumbent position, with the neck rotated in a contralateral direction to the vein examined, without straining the sternocleidomastoid muscle. Initially, one of the students conducted the ultrasound examination of both IJVs of the patient, using the PoCUS compression technique. After the student evaluation, each patient was also evaluated by an experienced physician, using the same compression technique. At the end of the session, an independent expert performed the color Doppler ultrasound technique of the IJVs for final confirmation. Each of the 3 operators was totally blind to the other examinations.

The compression technique was performed using the bidimensional ultrasonographic mode, with the transducer that was moved (sliding) in a transverse view from the crossing point of the two portions of the sternocleidomastoid muscle down to the clavicle, to evaluate the entire extent of the IJV. During the movement of the probe, compression maneuvers were continuously attempted along the whole course of the vessel, pressing the transducer in an anteroposterior direction to obtain collapse of the lumen (Additional file 1: Video S1).

The compression maneuver was considered positive for thrombosis in two conditions: (1) visualization of a hyperechogenic image inside the vein, without the possibility to collapse the lumen (Additional file 2: Video S2); (2) even in the absence of any image inside the lumen, absence of fully collapse under the application of a sufficient force that anyway could not be enough to deform the adjacent artery (Additional file 3: Video S3). In case of a persisting suspicion without the possibility for final confirmation, a further compression maneuver was performed with the transducer positioned longitudinally to the vessel (Additional file 4: Video S4). This maneuver was never repeated more than once to reduce the risk of embolism.

The color Doppler technique requires a higher skill level. In the present study we consider the total or partial absence of blood flow in the color Doppler examination as an indicative of vein thrombosis and we used it as the gold standard for the confirmation of the diagnosis (Additional file 5: Video S5).

All the exams were registered, and the clips were stored.

#### Ethical aspects

The Informed Consent Form was signed by the study participants or by their legally authorized representative. The study was approved by the Research Ethics Committee of FAME Medical School (Consolidated Opinion 3.678.861).

### Statistical analysis

The statistical analysis was processed by Stata software, version 9.2 (StataCorp, College Station, TX, USA). Accuracy, sensitivity, specificity, positive predictive value, negative predictive value and the respective confidence intervals were calculated. The Kappa statistical method, proposed by Jacob Cohen, was used with the objective of measuring the agreement between proportions derived of dependent samples. The interpretation of the Kappa results was conducted according to the study of Silva RS and Paes AT in 2012, who described different values according to the degree of agreement suggested [13]. Differences were considered significant when *p* < 0.05.

## Results

The demographic data, family history and comorbidities of the 81 enrolled patients are detailed in Table 1.

**Table 1:** Descriptive analysis of demographic data and comorbidities of the sample

Variables	
Demographic data	
Gender (male)—n (%)	51 (62.96)
Age (average, in years)	56.2 (SD: 16.7)
BMI—n (%)	80
Underweight	7 (8.75)
Adequate weight	44 (55.00)
Overweight	18 (22.50)
Obese	11 (13.75)
Comorbidities—n (%)	
Hypertension	61 (78.21)
Diabetes	29 (35.80)
History of previous neoplasia	6 (7.69)
Family history—n (%)	
Neoplasia (up to first-degree relatives)	23 (29.49)

Pró-Renal Hemodialysis Clinic database; *SD* Standard Deviation, *BMI* Body Mass Index (categorization source: Center for Diseases Control)

We evaluated 162 internal jugular veins of 81 patients. The results obtained by the students and expert physicians using the compression technique, were compared to the results obtained with the use of color Doppler (Table 2).

**Table 2:** Comparison of the results of the evaluation of TR-CVCd by compression technique in relation to the color Doppler technique

	Trained medical students		PoCUS expert physicians	
	%	IC (95%)	%	IC (95%)
Sensitivity	59.2	51.6–66.8	100	100
Specificity	89.6	84.9–94.3	94.8	91.4–98.2
PPV	53.3	45.6–61.0	79.4	73.2–85.6
NPV	91.6	87.3–95.9	100	100
Accuracy	84.6	_	95.7	_

The compression technique showed sensitivity of 59.2% when performed by students (CI 51.6–66.8%) and 100% when performed the experts. The specificity was of 89.6% in the group of students (CI 84.9–94.3) and 94.8% (CI 91.4–98.2) in the group of experts. Positive predictive value and negative predictive value were 53.3% (CI 45.6–61.0) and 91.6% (CI 87.3–95.9) for the exams performed by students and 79.4% (CI 73.2–85.6) and 100% when performed by experts.

The average timing of catheter-day was 328 in the group of patients with TR-CVCd and 115 in the groups without thrombosis (*p* = 0.0311).

## Discussion

The number of patients in hemodialytic therapy and with a consequent need for CVCd has grown worldwide. TR-CVCd is a common complication and may have negative consequences that vary from simple catheter disfunction, which compromises the dose of dialysis offered, up to potentially fatal complications, such as pulmonary embolism [9, 14].

In the present study, the ultrasound compression technique was compared to the color Doppler technique in the diagnosis of TR-CVCd. We used the evaluation by Doppler as the gold standard exam, based on a previous study that demonstrated an excellent accuracy of this technique when compared to venography [15].

The prevalence of TR-CVCd in the IJV found in our population was similar to that of previous studies [16, 17]. The average time of catheter use was of 328 catheter-day among patients with TR-CVCd and 115 catheter-day in patients without TR-CVCd, which demonstrated a clear correlation between the time of the CVCd permanence and the occurrence of thrombosis. This finding is troubling, as we consider that in the United States, for instance, 25% of the patients in hemodialysis do the treatment through a CVCd.

We demonstrated in a previous publication that medical students can be trained to recognize the major cervical vessels using PoCUS, after a short focused training [18]. In the current study, the compression technique performed by medical students demonstrated an inferior diagnostic accuracy when compared to that performed by expert physicians. The compression PoCUS in the hands of experts demonstrated highly accurate for the thrombosis of the IJV. Prandoni and collaborators compared the compression technique to venography (gold standard) and found sensitivity and specificity of 96% and 93.5%, respectively, quite similar results to those obtained in our study [15].

The lower sensitivity of the test obtained by medical students can be explained by the fact that the majority of thrombi were present in the portion of the IJV closer to the subclavian vein where IJV combines with the subclavian vein to form the brachiocephalic trunk. Indeed, the presence of thrombus in that location increases the complexity of the examination, because the correct visualization depends on a proper angulation of the transducer for the insonation of the supraclavicular fossa, while for an ideal compression technique, the probe should be placed in a strict perpendicular position. That is not always an easy maneuver to acquire from the beginners. This technical aspect for scanning this specific region was not emphasized during the training performed by the students. However, despite a short and probably incomplete teaching, the students were able to assign the diagnosis with good specificity, quite similar to that reported in the literature and to the performance of experienced physicians involved in the present study [15]. Moreover, the high negative predictive value achieved by experts and also by students has important clinical meaning since ruling out thrombosis is the target of the one in charge of cannulating the vein for CVCd.

Regarding the application of a simplified PoCUS compression technique, it is important to emphasize the striking difference in complexity when compared to the Doppler examination. The learning curve of the compression technique is quite steep and opens the possibility to train operators who are not experts in ultrasound. Moreover, the compression maneuver does not need sophisticated software and can be performed also using basic and cheap machines at bedside. Our data show that the compression technique is reliable especially to rule-out TR-CVCd in the IJV when it is performed by experienced personnel. Thus, this technique may become the standard of care in centers dedicated to dialytic patients, without the need for more advanced ultrasound resources [19].

This study has some limitations. For the convenience of the patients, all the examinations were performed during their dialysis sessions, with the patients in semi recumbent position. Indeed, the supine position would be ideal for the insonation of cervical vessels and probably increases the reliability of the compression maneuver especially for non-expert operators. In addition, the majority of examinations were performed in the final third during the time course of the dialysis sessions, when the patients had already been subjected to varied levels of ultrafiltration. Therefore, our population was studied in a state of relative depletion of the intravascular volume, which has probably an impact in reducing the optimal visualization of the vein lumen and may have influenced the low sensitivity obtained by the non-experienced operators.

It is also important to recognize that more studies with larger number of patients and students are necessary to corroborate the results. Furthermore, what to teach and how to teach students to be as accurate as well trained persons needs more research.

## Conclusion

This study demonstrated that the PoCUS compression technique for the diagnosis of TR-CVCd in the IJV is reliable and accurate when performed by expert physicians in comparison to the Color Doppler venous examination. Despite the small sample of students in this study, it is possible to conclude that a training program to teach the compression technique to non-expert students and form new operators is feasible, but needs to be optimized and more detailed to reach a sufficient level of efficiency. Finally, our study demonstrated that the TR-CVCd of the IJV has a high prevalence in our traditional dialysis center. This high prevalence maybe expected also in other centers and should be carefully investigated. An efficient training of nephrologists to the use of PoCUS would allow a more diffuse early diagnosis of this complication, which is crucial to avoid all the possible deleterious consequences of the misdiagnoses.

## Supplementary Information

**Additional file 1: Video S1.** Image of the internal jugular vein being collapsed during the sliding of the probe and the compression technique.

**Additional file 2: Video S2.** Thrombosis of the internal jugular vein diagnosed by the visualization of a hyperechogenic image inside the vein, without the possibility to collapse the vessel.

**Additional file 3: Video S3.** Thrombosis of the internal jugular vein diagnosed by the absence of fully collapse under the compression technique even in the absence of any hyperechogenic image inside the lumen.

**Additional file 4: Video S4.** Compression maneuver carried out with the transducer positioned longitudinally to the vessel to confirm the hypothesis of thrombosis.

**Additional file 5: Video S5.** Presence of flow by color Doppler technique inside the internal jugular vein.

## Data Availability

Availability of data is not applicable to this article.

## Abbreviations

CVCd: Central venous catheter for dialysis
IJV: Internal jugular vein
PoCUS: Point of care ultrasound
TR-CVCd: Thrombosis related to the central venous catheter for dialysis

## References

[1] Levin A, Tonelli M, Bonventre J, Coresh J, Donner J, Fogo AB, Fox CS, Gansevoort RT, Heerspink HJL, Jardine M, Kasiske B, Köttgen A, Kretzler M, Levey AS, Luyckx VA, Mehta R, Moe O, Obrador G, Pannu N, Parikh C, Perkovic V, Pollock C, Stenvinkel P, Tuttle KR, Wheeler DC, Eckardt KU (2017). A roadmap for closing gaps in care, research, and policy. Lancet.

[2] Linardi F (2013). The importance of vascular access for hemodialysis in Brazil. J vasc bras.

[3] Ethier J, Mendelssohn DC, Elder SJ, Hasegawa T, Akizawa T, Akiba T, Canaud BJ, Pisoni RL (2008). Vascular access use and outcomes: an international perspective from the Dialysis Outcomes and Practice Patterns Study. Nephrol Dial Transplant.

[4] Lok CE (2007). Fistula first initiative: advantages and pitfalls. Clin J Am Soc Nephrol.

[5] Lok CE, Foley R (2013). Vascular access morbidity and mortality: trends of the last decade. Clin J Am Soc Nephrol.

[6] Agraharkar M, Isaacson S, Mendelssohn D, Muralidharan J, Mustata S, Zevallos G, Besley M, Uldall R (1995). Percutaneously inserted silastic jugular hemodialysis catheters seldom cause jugular vein thrombosis. ASAIO J.

[7] Karnik R, Valentin A, Winkler WB, Donath P, Slany J (1993). Duplex sonographic detection of internal jugular venous thrombosis after removal of central venous catheters. Clin Cardiol.

[8] Lee JA, Zierler BK, Zierler RE (2012). The risk factors and clinical outcomes of upper extremity deep vein thrombosis. Vasc Endovascular Surg.

[9] Gunawansa N, Sudusinghe DH, Wijayaratne DR (2018). Hemodialysis catheter-related central venous thrombosis: clinical approach to evaluation and management. Ann Vasc Surg.

[10] Nunes AA, PazeliJúnior JM, Rodrigues AT, Tollendal AL, Ezequiel Oda S, Colugnati FAB, Bastos MG (2016). Development of skills to utilize point-of-care ultrasonography in nephrology practice. J Bras Nefrol.

[11] Hassanzadeh Rad A, Badeli H (2017). Point-of-care ultrasonography: is it time nephrologists were equipped with the 21th century’s stethoscope?. Iran J Kidney Dis.

[12] Niyyar VD, O’Neill WC (2018). Point-of-care ultrasound in the practice of nephrology. Kidney Int.

[13] Silva RS, Paes AT (2012). Por dentro da estatística. Educ Contin Saúde Einstein.

[14] Sehgal AR, Snow RJ, Singer ME, Amini SB, DeOreo PB, Silver MR, Cebul RD (1998). Barriers to adequate delivery of hemodialysis. Am J Kidney Dis.

[15] Prandoni P, Polistena P, Bernardi E, Cogo A, Casara D, Verlato F, Angelini F, Simioni P, Signorini GP, Benedetti L, Girolami A (1997). Upper-extremity deep vein thrombosis. Risk factors, diagnosis, and complications. Arch Intern Med.

[16] Yardim H, Erkoc R, Soyoral YU, Begenik H, Avcu S (2012). Assessment of internal jugular vein thrombosis due to central venous catheter in hemodialysis patients: a retrospective and prospective serial evaluation with ultrasonography. Clin Appl Thromb Hemost.

[17] Wilkin TD, Kraus MA, Lane KA, Trerotola SO (2003). Internal jugular vein thrombosis associated with hemodialysis catheters. Radiology.

[18] Pazeli JM, Vieira ALS, Vicentino RS, Pazeli LJ, Lemos BC, Saliba MMR, Mello PA, Costa MD (2018). Point-of-care ultrasound evaluation and puncture simulation of the internal jugular vein by medical students. Crit Ultrasound J.

[19] Brass P, Hellmich M, Kolodziej L, Schick G, Smith AF (2015). Ultrasound guidance versus anatomical landmarks for internal jugular vein catheterization. Cochrane Database Syst Rev.

